# Reproducibility in Carbon Nanotube-Based Hydrogels: The Role of CNT Material State and Reporting

**DOI:** 10.3390/gels12040273

**Published:** 2026-03-26

**Authors:** Elsa Gabriela Ordoñez-Casanova, Rosa Alicia Saucedo-Acuña, Karla Lizette Tovar-Carrillo, Hector Alejandro Trejo-Mandujano

**Affiliations:** 1 Instituto de Ingeniería y Tecnología, Universidad Autónoma de Cd. Juárez, Av. del Charro y Henry Dunant s/n, Omega, Ciudad Juárez 32584, Chihuahua, Mexico; 2 Instituto de Ciencias Biomédicas, Universidad Autónoma de Cd. Juárez, Av. Benjamín Franklin # 4960, Zona Pronaf, Ciudad Juárez 32315, Chihuahua, Mexico; rosauced@uacj.mx (R.A.S.-A.); karla.tovar@uacj.mx (K.L.T.-C.)

**Keywords:** carbon nanotubes, hydrogels, material comparability, reporting completeness, material traceability, nanomaterial characterization, dispersion

## Abstract

Carbon nanotube (CNT)-based hydrogels continue to present a persistent challenge of material comparability, as systems that appear equivalent frequently generate different mechanical, electrical, and biological responses. Although experimental variability is frequently cited as the primary explanation, many discrepancies arise from comparing systems whose nanotubes differ structurally in ways that are rarely documented. Diameter distribution, defect density, residual catalyst content, and surface chemistry directly influence CNT dispersion, network integration, and interactions in hydrated polymer matrices. When these parameters are insufficiently reported, formulations that appear comparable may represent materially distinct systems. In this review, the CNT–hydrogel literature is reconsidered from the perspective of material comparability. Rather than focusing only on whether reported results agree across studies, this review evaluates whether sufficient structural and processing information is available to determine if the systems being compared are materially equivalent. Selected publications were analyzed using a reporting-based descriptor framework encompassing nanotube origin, structural characterization, dispersion, microstructure, transport behavior, and biological relationships. A consistent pattern emerges: reproducibility becomes more interpretable when nanotube identity and processing history are documented with sufficient resolution. This enables meaningful cross-study comparison without requiring strict protocol standardization.

## 1. Introduction

Reports on CNT–hydrogel systems often present comparable CNT loadings and polymer matrices [1]. However, the resulting mechanical and electrical properties are not always consistent across laboratories [2,3,4,5,6,7]. These differences are often attributed to variations in experimental handling or measurement protocols. However, even when the preparation steps and evaluation methods appear similar, discrepancies in stiffness or conductivity may persist. Under such circumstances, it becomes difficult to determine whether the variation arises from procedural factors or structural differences in the nanotube populations incorporated into each system. Consequently, simply repeating a formulation and comparing measured properties provides an incomplete assessment of reproducibility in CNT-based hydrogels [8,9]. Carbon nanotube-reinforced hydrogels have attracted attention because they combine the hydrated compliance of polymer networks with the functional attributes of nanocarbon structures [2,3,10]. This broader technological interest in CNTs has also accelerated the diversification of application-driven CNT composite systems [11]. As a result, CNT–hydrogels have been explored for soft electrodes, tissue scaffolds, strain sensors, and other biointegrated devices [5,12,13,14,15,16,17,18,19,20]. Recent studies on hierarchical carbon nanotube assemblies further illustrate how nanotube architecture and cross-linking strategies can significantly influence conductivity and multifunctional performance in CNT-based materials [21], highlighting the strong dependence of macroscopic properties on nanotube structure and processing history.

In these systems, CNTs are not passive additives: once incorporated into a hydrated network, they shape local conductivity and interfacial organization and, under certain conditions, form percolating pathways that govern the macroscopic behavior of the gel [15,22,23]. Their stiffness and chemical stability further support these multifunctional roles [24,25,26].

Despite this promise, reports describing similar CNT loadings and polymer matrices often reach dissimilar conclusions regarding their stiffness, conductivity, and cytocompatibility [2,3,4]. Experimental variability has frequently been invoked to explain these discrepancies [8,9]. However, this explanation assumes that the nanotubes used in different laboratories are effectively interchangeable. This assumption is not trivial for materials whose diameter distribution, defect density, residual catalyst content, and surface chemistry depend strongly on the synthesis and post-treatment history [24,25,26].

Biological evaluation highlights this issue more clearly. Experimental studies on CNT-containing systems report that changes in aggregation state or surface chemistry can shift cellular responses, even when bulk compositions appear comparable [27,28,29]. Within hydrogels, dispersion quality and polymer–nanotube interactions can either limit nanotube accessibility or create localized regions with higher exposure [30]. This is consistent with evidence that biomolecular adsorption at nanomaterial interfaces can modulate biological outcomes [31]. Therefore, two systems with the same nominal composition may still present different biological interfaces. Many studies describe nanotube synthesis and structure only in broad terms [2,12,13], with limited experimental linkage between these features and the resulting hydrogel network. Because hydrogels reorganize around the physicochemical identity of their fillers [26,32,33,34], synthesis-dependent differences in defect density or residual metal content can alter nanotube geometry and interfacial interactions and, in turn, produce measurable shifts in mechanical and electrical properties [32,33,34]. When these parameters are not reported with sufficient resolution, differences between studies become difficult to interpret [8,9].

In many CNT–hydrogel studies, important details regarding nanotube characterization and processing are only described or excluded completely. This deficit of specificity complicates experimental replication and makes it difficult to determine whether results reported by different groups are genuinely comparable [8,9]. Systems that appear similar on paper may in fact be based on nanotubes with distinct structural features, purification histories, or dispersion states, leading to differences that are not immediately evident from composition alone.

Clarity in reporting synthesis origin, structural characteristics, dispersion conditions, and internal microstructure is therefore essential for interpreting functional outcomes. Carbon nanotubes are not interchangeable components; variations introduced during synthesis and post-treatment leave structural traces that influence how they integrate within polymer networks [1,2] and how the resulting hydrogels perform.

This review examines how nanotube definition, preparation history, and microstructural verification influence the interpretation of mechanical, electrical, and biological outcomes across the CNT–hydrogel literature. Many inconsistencies attributed to setups or testing conditions may instead reflect incomplete material definition and limited traceability, particularly when non-commercial or laboratory synthesized CNTs are used. By analyzing reporting patterns through a consistent form framework, this work highlights the material level information needed to support significant structure–property comparisons between studies. The objective of this review is to examine CNT–hydrogel systems from the perspective of material comparability, identifying how nanotube definition, processing history, and reporting completeness influence the interpretation of structure–property relationships across the literature.

## 2. Literature Selection and Analytical Approach

This review examines how carbon nanotube-based hydrogels are described when functional performance is reported. Rather than attempting to catalogue every formulation, the analysis focuses on whether studies provide sufficient information about nanotube identity and processing history to connect the material to its mechanical, electrical, or biological behavior. Relevant publications from 2006 to 2025 were identified through database searches using terms related to carbon nanotubes, hydrogels, dispersion, and cytotoxicity. Only studies reporting functional performance together with at least some physicochemical characterization and processing details were included. Application-oriented reports lacking material information were excluded, since they do not allow evaluation of material comparability. To highlight reporting patterns, each study was assessed using a set of descriptors covering CNT source, structural characterization, dispersion conditions, microstructure, transport behavior, and biological linkage. The studies included in Table 1 were chosen as representative examples across different publication years, application areas, and levels of reporting detail to highlight common reporting patterns rather than to comprehensively cover the field.

These studies are not intended to represent a systematic review of the field, but rather illustrative examples used to highlight how differences in reporting practices affect cross-study interpretability. The goal is not to rank individual works or to provide a field-wide quantitative assessment, but to illustrate how differences in reporting influence the ability to compare materials across studies. The descriptors summarized in Table 1 therefore serve as a practical framework for identifying which aspects of nanotube definition and processing are consistently documented and which are often omitted.

A reporting completeness score was calculated as the fraction of descriptors explicitly reported in each study (0–1 scale), as shown in Table 1. This score is intended to reflect how transparently the material and processing details are described, and therefore how easily the study can be interpreted and compared with others. It does not aim to evaluate experimental rigor, study quality, or the validity of the reported results. While the scoring was based on defined criteria, some level of interpretation is inherent in this type of analysis. In addition, variations in reporting practices over time may also influence the observed differences. It is also important to distinguish material comparability from reproducibility or replicability, which depend on additional factors such as experimental procedures and measurement conditions.

## 3. Carbon Nanotube-Based Hydrogel Networks: Current Practices and Limitations

Application-driven research has accelerated the development of CNT–hydrogel systems considerably [13,36]. However, it has also exposed a persistent problem: similar systems frequently produce different results. This stems from the fundamental differences between conventional CNT-reinforced composites and hydrogel networks. In solid composites, nanotubes primarily function as mechanical and electrical reinforcements within a stable matrix, and their performance is interpreted accordingly [24]. In hydrogels, the polymer–water environment is far more sensitive [13,37]. Interfacial and transport effects become significant, and the physicochemical identity of the nanotubes directly shapes the formation and behavior of the network [26].

However, this sensitivity is rarely reflected in the characterization. Most CNT–hydrogel studies identify nanotubes using broad labels such as multiwall carbon nanotubes (MWCNTs), while leaving defect density, catalyst residues, aggregation state, and surface chemistry unreported [24,25,26]. This is important because evidence from conductive hydrogel research consistently shows that filler selection and processing, not just composition, determine electrical stability, mechanical performance, and long-term functionality, particularly in biointegrated applications, where small material differences produce measurable performance shifts [6,13,15,22,35,36,38,39].

The dispersion further complicates this problem. Polymer-assisted stabilization is a standard practice, but it is rarely described with sufficient quantitative detail to allow replication or comparison, even though the dispersion state directly influences percolation, mechanical response, and local heterogeneity within the gel [32,33]. Gel formation depends not only on the CNT concentration but also on the nanotube geometry, dispersant compatibility, and processing history factors, which can produce fundamentally different network states, including reversible gelation, from nominally identical starting points [4,7].

**Figure 1:**
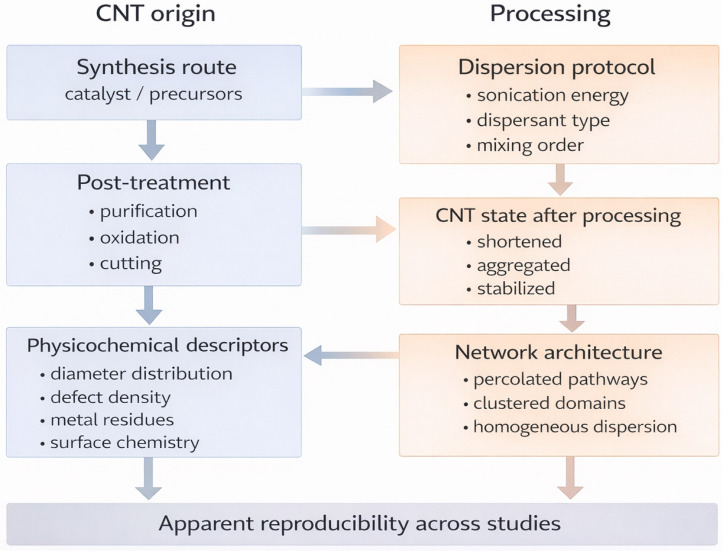
Conceptual illustration showing how nanotube origin, structural characteristics, and processing conditions influence dispersion, network formation, and ultimately the mechanical, electrical, and biological properties of CNT–hydrogel systems. The diagram highlights that when these aspects are not clearly reported from the outset, material traceability becomes limited, making it difficult to distinguish true experimental variability from differences related to the nanotube material state.

This is because hydrogels do not simply contain nanotubes; they reorganize around them. The surface energy, defect density, and residual catalyst particles alter polymer adsorption, water structuring, and interfacial confinement, generating distinct percolation pathways and transport domains, even at identical compositions [26,32,33]. These structural differences are rarely captured in bulk measurements; however, they influence both long-term stability and biological interactions. Studies on electrophysiological electrodes have clearly demonstrated that the signal quality correlates more strongly with the microstructure and integration strategy than with the nominal composition [15,38]. As illustrated in Figure 1, insufficient definition at early stages of nanotube selection and processing can propagate into the resulting hydrogel structure and performance, making apparent irreproducibility difficult to interpret.

Safety assessments also reflect this gap in material definition and reporting, particularly when advanced tools and context-aware interpretations are not applied [40]. The biological outcomes in CNT-containing systems depend on the filler chemistry and dispersion state; however, reports on both remain uncommon [27,28,29]. The result is a body of literature where traceability is limited, dispersion is underreported, and safety data cannot be meaningfully connected to material identity, three conditions that collectively undermine data reproducibility.

This pattern does not appear to be random. Across the studies examined here, differences in reported outcomes often coincide with differences in reporting completeness rather than with experimental error alone. To illustrate this point, representative publications were evaluated according to how clearly they described the nanotube identity, processing conditions, and structural verification. The resulting score reflects reporting-based interpretability, that is, how easily the findings of a study can be compared with those reported by others based on explicitly documented material and processing information, rather than the intrinsic performance of the material system itself.

For this analysis, each descriptor was assigned a binary value (1 when the information was explicitly reported and 0 when it was not). A value of 1 required that the reported information be sufficiently specific to support independent material-level interpretation or cross-study comparison. For the descriptor ‘CNT source defined’, a value of 1 was assigned only when the study provided traceable information on nanotube origin, either through supplier/commercial identification or through description of the synthesis route together with relevant purification, functionalization, or post-treatment history. When this level of detail was not available, a value of 0 was assigned. The reporting completeness score was then calculated as the fraction of descriptors reported relative to the total number considered.

The score shown in Table 1 reflects how clearly each study describes the nanotube material and the way it was processed, both of which are important for comparing results across literature. In this context, the score is intended to indicate how easily a study can be interpreted and compared with others, rather than evaluate the intrinsic performance, methodological rigor, or scientific quality of the hydrogel system.

Across the evaluated studies, reporting completeness was highly variable. The mean reporting completeness score was 0.41, with values ranging from 0.125 to 0.75, indicating substantial heterogeneity in the documentation of CNT identity and processing history. Although structural marks such as Raman characterization were commonly included, parameters directly influencing material comparability, particularly residual metal content and quantified dispersion conditions were reported far less consistently. This irregular transparency suggests that similar CNT–hydrogel systems may in fact rely on materially distinct nanotube populations, complicating cross-study comparison and interpretation of performance or safety outcomes [8,9]. This pattern supports the view that at least part of the apparent inconsistency across CNT–hydrogel studies may arise from incomplete material traceability rather than from experimental variability alone.

## 4. Dispersion and Interfacial Interactions in CNT–Hydrogel Networks

Dispersion is more than a preparatory step. It is the point at which the nanotube identity begins to express itself structurally in the hydrogel [26,32,33]. Because CNTs exhibit strong van der Waals interactions and hydrophobic surface characteristics, they tend to bundle in aqueous environments [26,37,41,42], and achieving stable separation requires balancing the nanotube properties, processing conditions, and polymer–nanotube compatibility [26,43]. The balance achieved during dispersion largely determines the resulting network architecture and functional behavior. Quantitative evaluation of dispersion state can be supported by complementary characterization techniques such as UV–visible spectroscopy, dynamic light scattering, or microscopy-based image analysis, which provide indirect or direct indicators of bundle fragmentation and nanotube distribution in suspension. These approaches also help document dispersion in a way that supports meaningful comparison across studies.

Ultrasonication combined with surfactants, polymers, or biomacromolecules is the standard approach for breaking up bundles, and it works. However, the outcome is sensitive to parameters that are routinely underreported, such as sonication energy, duration, temperature, and dispersant chemistry [32,33]. Small differences in any of these factors can produce different dispersion states from nominally identical starting materials, making replication difficult even when the written protocol appears complete. Colloidal stability is a central consideration in nanocarbon processing across systems and media [44].

Once the nanotubes are dispersed, their stability within the network depends on the extent of the interaction between the polymer chains and their surface. Wrapping, hydrogen bonding, and electrostatic interactions all contribute, but their effectiveness is not uniform; it varies with the surface chemistry, defect density, and residual catalyst species [26,30,45]. Therefore, two nanotube samples with similar dimensions but different synthesis histories can be organized very differently within the same polymer matrix, producing distinct network architectures from what appear to be the same formulation.

At the microstructural level, these differences are significant. Well-dispersed CNTs establish interconnected pathways that mechanically reinforce the matrix and support electrical percolation [13,38], whereas aggregated CNTs produce CNT-rich clusters embedded in polymer-dominated domains [13,23,34,38]. These structural differences influence the mechanical response, electrical transport, and cell interactions in ways that bulk measurements may not capture and that nominal compositions alone cannot predict.

However, key descriptors, such as nanotube diameter, defect density, and dispersion conditions, are frequently absent from published reports [26,30]. CNTs obtained from different synthesis sources often carry characteristic defects or catalyst residues that affect their coupling with the polymer and their behavior during gelation [46,47]. However, these details rarely appear in performance data.

Taken together, the dispersion state and interfacial chemistry are not peripheral variables; rather, they are the mechanisms by which the nanotube identity translates into hydrogel behavior. A shift in the processing sequence or a minor variation in the preparation conditions can change a system from a relatively uniform network to a highly heterogeneous one. When this occurs without documentation, the outcome tends to be logged as experimental variability rather than being recognized as a material effect. Therefore, treating dispersion and interfacial interactions as primary reporting variables rather than procedural footnotes is a practical step toward more interpretable CNT–hydrogel research and directly supports the minimum descriptors needed for material comparability.

## 5. Reproducibility and Safety as Functions of Nanotube Material State

Many outcomes described as reproducibility failures in CNT–hydrogel systems reflect limited material comparability rather than true experimental inconsistency. Nanotube diameter distribution, defect density, residual catalyst content, surface chemistry, and aggregation tendency are largely determined by synthesis and post-treatment history. When nanotubes with different structural histories are incorporated into nominally identical formulations, the internal architecture of the hydrogel network may change even if polymerization conditions are held constant. In such cases, apparent contradictions arise not from flawed methodology but from differences in the material state of the nanotubes themselves [24,25,26].

Processing history introduces an additional layer of variability. Sonication energy, dispersant identity, and mixing sequence influence nanotube length distribution, dispersion stability, and percolation threshold [32,33,34]. Because these parameters are often reported in general terms, two laboratories may follow similar written protocols yet generate networks with distinct internal architectures. Nominal compositional equivalence does not guarantee structural equivalence, and differences in mechanical or electrical behavior may therefore reflect microstructural divergence rather than inconsistent experimentation [8,9,13,38].

Biological response further illustrates this dependency within a defined physicochemical context [48]. Cellular interactions are shaped by aggregation state, surface defects, residual catalyst species, and nanotube accessibility within the hydrogel matrix [27,28,29,49,50]. Two systems with identical bulk compositions but different dispersion states present distinct exposure scenarios. Evidence suggests that metal speciation and surface chemistry can influence cytotoxicity more strongly than total metal content alone [29,49,50], underscoring the need to interpret safety data within a defined physicochemical context.

Reproducibility and safety in CNT–hydrogel systems are closely connected. Both depend on how clearly the nanotube material state is defined and documented. When nanotube origin, structural descriptors, and processing conditions are described with sufficient detail, differences in performance across studies become easier to interpret as consequences of distinct material histories rather than as unexplained variability. In this context, the key question is not simply whether two studies report similar outcomes, but whether the materials being compared are truly equivalent in terms of their nanotube characteristics and processing history. The interpretative implications of these material descriptors are summarized in Table 2, while they are further organized into a tiered framework later in the manuscript to support more consistent material comparability across studies.

**Figure 2:**
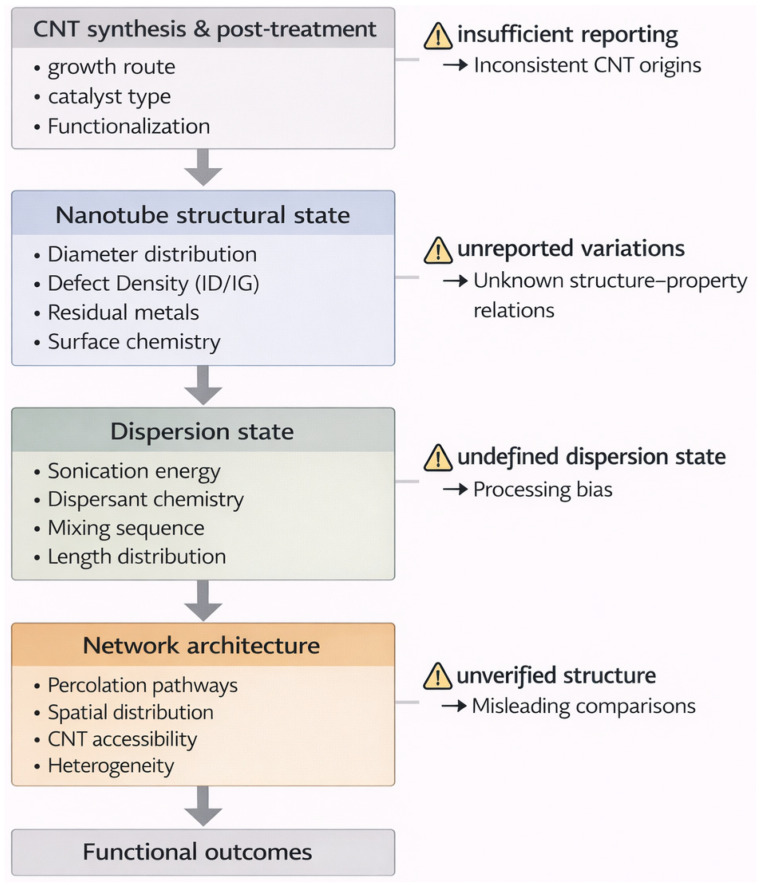
Schematic representation of traceability flow in CNT–hydrogel systems. The diagram illustrates how nanotube origin, processing history, dispersion state, and network architecture influence the interpretation of functional outcomes and where incomplete reporting may lead to divergent interpretations.

A schematic representation of traceability flow and interpretative difference in CNT–hydrogel systems is shown in Figure 2.

## 6. Toward More Reproducible CNT–Hydrogel Research: Practical Considerations

Improving reproducibility in CNT–hydrogel research does not require universal protocol standardization [8,9]. It requires material traceability. Studies should provide sufficient information about nanotube origin, structural forms, dispersion conditions, and resulting microstructure to allow meaningful comparison across laboratories [51].

Identification of the nanotubes is the starting point. Labels such as MWCNTs or SWCNTs, even when accompanied by a supplier name, describe the nanotube type but not its structural characteristics. Synthesis route and post-treatment history influence defect density, residual catalyst content, surface chemistry, and aggregation behavior, all of which affect dispersion, polymer coupling, and biological response [24,25,26,52]. Reporting this information allows readers to evaluate whether materials across studies are genuinely comparable.

Structural characterization should extend beyond nominal purity values [36,53,54]. Raman ID/IG ratios, approximate diameter and length ranges, representative microscopy images, and documentation of metal content provide essential context for interpreting functional outcomes [26,27,28,29]. Without these descriptors, performance data cannot be confidently linked to specific nanotube properties.

Dispersion parameters also deserve explicit reporting. Sonication energy, duration, dispersant chemistry, and processing sequence directly influence network architecture and percolation behavior [32,33,34]. Because these factors modify the effective material state, they should be treated as defining variables rather than as procedural details.

Microstructural verification closes the gap between formulation and function. Imaging approaches such as SEM, cryo-SEM, or confocal microscopy allow the spatial distribution of CNTs within the hydrogel to be correlated with mechanical, electrical, and biological behavior [34]. Bulk property measurements alone cannot distinguish between homogeneous networks and clustered domains.

When these material descriptors are reported consistently, differences in performance across studies become easier to interpret rather than appearing contradictory. In this context, reproducibility depends largely on how clearly the nanotube material state is defined and documented, rather than on enforcing identical experimental protocols. As illustrated in Table 3, this perspective shifts the focus away from minor compositional similarities and toward understanding CNT–hydrogel systems as distinct material systems whose physicochemical history shapes their behavior.

Table 3 summarizes the conceptual shift from treating CNTs as interchangeable additives to recognizing them as defined material systems. To make this perspective more practical, material traceability can be expressed through a small set of descriptors that help determine whether CNT–hydrogel systems are genuinely comparable.

These descriptors are organized into two levels in Table 4. Tier 1 represents the minimum information required to establish basic material comparability across studies. Tier 2 includes additional descriptors that strengthen mechanistic interpretation and safety assessment, particularly when outcomes depend on interfacial chemistry, hydrated-state architecture, or exposure-related effects. Rather than proposing strict reporting rules, the aim is to provide a practical reference that supports clearer comparisons across studies [24,25,26,27,28,29,32,33,34,55].

In practice, implementing these reporting recommendations is not always straightforward. Access to characterization tools varies across laboratories, and some descriptors—such as impurity speciation, hydrated-state imaging, or long-term release behavior—may require specialized instrumentation. Space limitations in journal articles can also limit how much detail can be reported. For this reason, the tiered structure proposed here is intended to remain flexible: Tier 1 defines the minimum information needed for basic material comparability, while Tier 2 includes additional descriptors that strengthen mechanistic interpretation and safety relevance when feasible.

Applying Tier 1 would already eliminate many comparisons between nominally similar but materially distinct CNT–hydrogel systems that are currently interpreted as “irreproducibility”. Tier 2 targets the situations where outcomes are governed by interfacial chemistry and the exposure scenario, which composition alone cannot capture [27,28,29,30].

In this framing, reporting is not administrative detail; it is the basis for cumulative, comparable evidence across laboratories and for separating true experimental variability from material-state effects [8,9].

## 7. Remaining Challenges and Open Questions

Despite substantial progress in CNT–hydrogel research, meaningful comparison across studies remains difficult. Nominal composition is routinely reported, yet dispersion quality, internal network organization, and interfacial structure are not consistently characterized using shared criteria [9]. Two materials that appear identical at the formulation level may represent distinct physical systems once integrated into a hydrated matrix.

This limitation becomes especially significant in biological evaluation. Cytocompatibility is often treated as an intrinsic property of the composite; however, cellular exposure depends on aggregation state, matrix stability, residual catalyst species, and potential release of surface-bound components [27,28,29]. Without shared physicochemical descriptors, it is not possible to determine whether a biological outcome reflects a material-dependent effect or uncontrolled variation.

Functional performance presents a parallel challenge. Electrical conductivity and mechanical stiffness are typically correlated with nanotube loading, yet similar formulations frequently yield divergent results. Available evidence suggests that internal network architecture plays a dominant role [23,34], but quantitative structure–property relationships remain insufficiently developed. Until these relationships are better established, performance comparisons will continue to rely on incomplete material context.

The central challenge, therefore, is not the replication of identical formulations but the establishment of shared criteria for material equivalence. Open questions remain regarding how dispersion state should be defined and quantified in a way that is comparable across laboratories, for example through combined use of spectroscopy, microscopy, and stability-related metrics [43,57]. A second challenge is how nanotube accessibility and spatial distribution within hydrated hydrogel matrices can be characterized without relying exclusively on dried-state observations [23,37,58]. Long-term structural and colloidal stability also remain insufficiently documented in most reports [55]. Progress in these areas will determine whether independent studies converge toward a cumulative understanding or continue to develop in parallel without a common interpretative framework [51]. Future progress will likely require collaborative efforts, including round-robin studies and shared characterization protocols, to evaluate how different nanotube populations behave within hydrated polymer networks under comparable conditions [59]. Addressing these challenges will require coordinated reporting practices and cross-laboratory validation strategies.

## 8. Conclusions

The analysis presented here reframes variability in CNT–hydrogel systems as a question of material comparability rather than experimental inconsistency. Carbon nanotubes are not interchangeable fillers; their synthesis history, structural forms, and processing conditions define the internal architecture of the hydrogel and shape its mechanical, electrical, and biological behavior.

When nanotube identity and processing parameters are insufficiently documented, nominally similar formulations may correspond to structurally distinct material systems. Under these conditions, performance differences across studies are difficult to interpret and are often attributed to experimental variability rather than to differences in material state.

Reproducibility in CNT–hydrogel research therefore depends on the clarity with which the nanotube material state is defined and reported. Transparent documentation of origin, structural characteristics, dispersion conditions, and microstructural organization provides the basis for meaningful cross-study comparison. Establishing such reporting practices does not require strict protocol standardization; it requires sufficient material traceability to determine when two systems are genuinely comparable. These recommendations are intended to complement, rather than replace, existing reporting and characterization guidelines for nanomaterials, including frameworks such as MIRIBEL and ISO/TR 13014:2012 [54], which emphasize transparent documentation of physicochemical properties [60].

## Figures and Tables

**Table 1:** Reporting completeness assessment of representative CNT–hydrogel studies based on key descriptors of nanotube identity, processing, and structure.

Reference	CNT Source Defined	Diameter/Length	Raman or Chemistry	Metal Impurities	Dispersion Quantified	Microstructure Imaged	Percolation Tested	Bio Linked to CNT	Reporting Completeness Score
Zhao et al. [2]	No	No	No	No	No	Yes	No	No	0.125
Huang et al. [3]	No	No	Yes	No	No	No	No	No	0.125
Zhu et al. [13]	No	No	No	No	No	No	Yes	No	0.125
Ogawa et al. [4]	Yes	Yes	Yes	No	Yes	Yes	Yes	No	0.75
Peñas-Núñez et al. [35]	No	No	No	No	No	No	Yes	No	0.125
Petersen et al. [28]	Yes	Yes	Yes	Yes	No	No	No	Yes	0.625
Donaldson et al. [27]	Yes	No	Yes	Yes	No	No	No	Yes	0.5
Jiang et al. [29]	Yes	No	Yes	Yes	No	Yes	No	Yes	0.625
Yu et al. [32]	Yes	Yes	Yes	No	Yes	No	Yes	No	0.625
Sabet et al. [33]	Yes	Yes	No	No	Yes	No	Yes	No	0.5

Note: Each descriptor was assigned a binary value (1 if the information was explicitly reported with sufficient detail to support material-level interpretation or cross-study comparison, and 0 if it was absent or insufficiently described). For the descriptor “CNT source defined”, a value of 1 was assigned only when the nanotube origin was explicitly traceable, either through supplier/commercial identification or through description of the synthesis route together with relevant purification, functionalization, or post-treatment history. Generic labels such as MWCNT or SWCNT alone were not considered sufficient. The reporting completeness score corresponds to the fraction of descriptors reported relative to the total number considered.

**Table 2:** Relationship between CNT material descriptors and interpretative consequences in hydrogel systems.

CNT Material Descriptor	Impact on Hydrogel Structure	Functional Implication	Risk If Not Explicitly Reported
Diameter distribution/aspect ratio	Governs percolation pathways and effective network connectivity	Directly affects electrical conduction and mechanical load transfer	Performance differences may be wrongly dismissed as laboratory-to-laboratory variability rather than material driven effects
Defect density (ID/IG)	Modulates interfacial interaction and polymer adsorption	Influences stiffness, stability, and transport behavior	Mechanical discrepancies may be misattributed to formulation changes instead of nanotube structural state
Residual catalyst content/metal speciation	Alters surface chemistry and potential release of reactive species	Modifies cytocompatibility and oxidative response	Systems with distinct impurity profiles may be interpreted as biologically equivalent
Aggregation state/dispersion quality	Controls spatial heterogeneity and CNT accessibility within the matrix	Produces divergent electrical pathways and biological exposure conditions	Bioresponse data become non-comparable despite apparently similar compositions
Sonication energy/processing sequence	Redefines effective length distribution and bundle fragmentation	Shifts percolation threshold and mechanical uniformity	Reported systems may not be structurally reproducible even if composition is identical
Microstructural verification (SEM, cryo-SEM, confocal)	Provides evidence of CNT spatial distribution	Enables meaningful structure–property correlation	Bulk properties risk being interpreted without confirmation of internal architecture

**Table 3:** Conceptual shift in CNT–hydrogel research: from generic additives to defined material systems.

Feature	Conventional Approach	Proposed Approach
Material identity	Reported by type and nominal purity only	Defined by synthesis route, catalyst residue, and structural fingerprints (ID/IG)
Role of processing	Treated as a neutral mixing step	Recognized as material defining, modifying aspect ratio, dispersion state, and network connectivity
Microstructure	Assumed homogeneous based on small loading	Network architecture correlated with performance using imaging
Safety interpretation	General conclusions (e.g., “the gel is biocompatible”)	Context specific assessment based on metal speciation and leaching potential
View of variability	Observed as experimental noise or biological complexity	Interpreted as a predictable result of distinct physicochemical histories

**Table 4:** Tiered reporting framework for assessing material comparability in CNT–hydrogel studies.

CNT Material Descriptor	Tier	Impact on Hydrogel Structure	Functional/Interpretative Implication	Key Refs
CNT source and history (supplier or synthesis route; purification/functionalization steps)	1	Defines the effective nanotube material state entering gelation	Enables traceability across studies; prevents treating distinct CNT populations as interchangeable	[24,25,26]
Dimensions (diameter range; approximate length/aspect ratio)	1	Controls network connectivity and transport pathways	Shifts percolation and reinforcement behavior even at similar loadings	[21,32,33,34,56]
Structural fingerprint (e.g., Raman ID/IG or equivalent descriptor)	1	Captures defect-related differences affecting interfacial coupling	Supports comparisons beyond generic SWCNT/MWCNT labels	[24,25,26]
Metal impurities (method + detection limit/threshold)	1	Alters surface chemistry and potential reactive species	Affects bioresponse interpretation; method/limit required for cross-study confidence	[27,28,29]
Dispersion conditions (dispersant identity/concentration; sonication energy/time or power/time)	1	Sets dispersion stability and effective length distribution	Strongly influences heterogeneity and percolation behavior	[32,33,34,56]
Microstructural verification (≥1 image: SEM/cryo-SEM/confocal)	1	Provides evidence of CNT spatial distribution within the gel	Prevents over-interpretation of bulk data without architecture confirmation	[34]
Impurity speciation/valence state (when feasible)	2	Determines reactivity and biological response pathways	Speciation can dominate cytotoxic outcomes beyond total metal content	[29]
Leachables/extractables under relevant conditions	2	Defines exposure scenario in hydrated networks	Anchors safety claims to accessibility and release, not bulk composition alone	[27,28,29,30]
Hydrated-state imaging (cryo-SEM or confocal)	2	Captures architecture in the hydrated state	Strengthens structure–property linkage under realistic conditions	[34]
Percolation/transport verification (threshold/connectivity test)	2	Confirms network-controlled macroscopic behavior	Distinguishes material-state effects from nominal composition effects	[34]
Aging/stability protocol (defined soaking/cycling/aging + re-test)	2	Reveals time-dependent structural and transport changes	Improves comparability for long-term functionality and safety interpretation	[55]

## Data Availability

No new data were created or analyzed during this study. Data sharing is not applicable to this study.

## Abbreviations

CNT	Carbon nanotube
SWCNT	Single-wall carbon nanotube
MWCNT	Multi-wall carbon nanotube
